# Lifestyle and Sociodemographic and Economic Characteristics of Patients with Lung Cancer in Morocco

**DOI:** 10.1155/2020/8031541

**Published:** 2020-01-06

**Authors:** Imane Harkati, Mohamed Kamal Hilali, Nezha Oumghar, Mouna Khouchani, Mohamed Loukid

**Affiliations:** ^1^Laboratory of Human Ecology, Semlalia Faculty of Sciences, Cadi Ayyad University, Marrakech, Morocco; ^2^Oncology Department, Center of Oncology and Hematology, Mohammed VI Hospital Center, Marrakech, Morocco

## Abstract

**Background:**

Lifestyle maintenance is a crucial condition before and after lung cancer disease. According to the previous research in the scientific databases, the effect of the interaction between socioeconomic and demographic factors on the lifestyle of lung cancer patients in Southern Morocco regions remains unexamined. Accordingly, this study was designed to examine the relationship between socioeconomic factors, demographic factors, and the lifestyle of lung cancer patients.

**Methods:**

A total of 133 patients with lung cancer were divided into 103 men and 30 women with a sex ratio of 3.43 and ages varying between 28 and 82 years, and they served as informants for the study and filled in a questionnaire to provide information on their sociodemographic background, various economic characteristics, and their lifestyle. These patients have also been submitted to an anthropometric examination following the standardized procedure recommended by the World Health Organization. The survey was conducted from July 2013 to March 2015 at the Oncology and Radiotherapy Department, at Mohammed VI Hospital Center in Marrakech, Morocco.

**Results:**

The preliminary results showed that the average age of patients was 59 ± 9 years. A proportion of 81% lived in the Marrakech-Safi region and 19% lived in four other southern regions. Among the patients, 6% were smokers, while 14% were nonsmokers and 80% were ex smokers. Following the discovery of the disease, 26% revealed that they had sleep disorders and 98% were reported to have a lack of appetite. Obesity, normal weight, and underweight were also taken as criteria to categorize the patients; thus, obese informants represented 23% of the total number, those having normal weight reached 67% and the patients having underweight represented 10%.

**Conclusion:**

Sociodemographic variables and various economic characteristics were shown to have a negative impact on the lifestyle of lung cancer patients.

## 1. Introduction

Most cancers in the world go undetected until the disease reaches an advanced stage, at which point the severe symptoms require diagnosis. Probably, the patients' low lifestyle due to their poor economic level cannot allow them to an easy attain for a priori diagnosis. Therefore, it is important to lead a higher lifestyle to have an easy access to clinical treatment and to attain significant survival rates [1, 2].

One man among five and one woman among six worldwide develop cancer during their lifetime. Moreover, one man among eight and one woman among eleven die of the disease [3]. The lifestyle of lung cancer patients is affected not only by their symptoms but also by their daily negative behavior [4, 5]. Normally, their daily life behavior resulting out of their low level of lifestyle leads to the increase of lung cancer risk [6, 7]. Being physically active might decrease the risk of lung cancer. On the contrary, consuming red meat, high body mass index, low fruit and vegetable intake, lack of exercise [8], and alcoholic drinks might increase the risk of lung cancer [9, 10].

In the United States, women die of more smoking-induced lung cancer than breast cancer. In some Nordic countries, lung cancer kills more women than men [6]. In fact, it is the first type of cancer among men in most Northern African Countries, notably Morocco with 31.9 per 100,000 [11].

In Morocco, lung cancer represents one of the major problems of public health. Indeed, the World Health Organization estimates that the annual number of new cases among men is 3,497. Besides, 12,500 men and 10,400 women die each year in the country [8]. The figures published on the incidence and mortality of cancer in Morocco are only estimations. In fact, there are only a few regional cancer records. For this reason, we conducted this epidemiological study to enrich the scientific and statistical knowledge on lung cancer in the southern region of the country.

The major problems of providing oncological care for patients with lung cancer in Morocco are the medical demography, the unequal distribution of professionals, and the health resources in the health regions of the country.

Generally, the great difficulty in accessing oncology care is due to the fact that patients are most often diagnosed at very advanced stages of the disease. At the national level, the diagnosis of lung cancer made at stage I or II attains only 4% of cases, while 96% of cases are diagnosed at stages III and IV [12]. Since the creation of Lalla Salma Foundation on November 22, 2005, the fight against cancer in Morocco has become a public health priority. To this end, the Foundation and the Ministry of Health have developed the National Plan for Prevention and Control of Cancer 2010–2019 [13].

Morocco's economic situation is developing at a rapid pace, and the standard of living is improving. This scenario has also resulted in sedentary lifestyles, unhealthy eating habits, smoking, alcohol consumption, urbanization, and the aging of the population. In this respect, our study aims at improving the management system and care for lung cancer patients and also developing all health professionals' importance of lifestyle outcomes in these patients.

The study will focus on monitoring changes in patients' weight related to cancer, dietary recommendations, exercise capacity, mental state, sleep habits, etc. In a context of etiological and analytical epidemiology, this study aims to examine the relationship between certain sociodemographic factors and the lung cancer patients' lifestyle in Southern Morocco.

## 2. Materials and Methods

### 2.1. Study Design

This study has a descriptive nature, applying a retrospective analysis of the data using anthropological and epidemiological approaches. A data collection about lung cancer disease was carried out for two years, starting from July 2013 until March 2015, applying a well-structured questionnaire on a sample of 133 patients (103 men and 30 women) at the center of oncology and hematology of the University Hospital Center of Marrakech. The study was conducted after approval of the protocol by “Mohamed VI Hospital Ethics Committee”; informants' consent was obtained in the study before data collection started.

The patients came from five different southern regions: Marrakech-Safi, Béni Mellal-Khenifra, Souss-Massa, Drâa-Tafilalet,, and Laâyoune-Sakia. These regions represent a number of 12,034,605 citizens, which is equivalent to 34.51% of the total Moroccan population. The survey was conducted by selecting a representative sample of the Moroccan Southern population through a purposive and quota sampling. The patients surveyed visit the center either for a first consultation, for a more precise follow-up of the evolution of the disease, or for serious health complications. The selection of patients was done in a structured way, respecting the inclusion criteria and the exclusion criteria. Accordingly, we have chosen patients who suffer from lung cancer, regardless of the stage and the cell type of the disease, while children and pregnant women together with patients having lung metastasis of another type of cancer were excluded.

Our sample is obviously representative because it is formed according to the purposive and the quota method through which we observe the criteria of sex, age, socioprofessional categories, region, etc. This sample that we studied represents the same characteristics as the southern population of Morocco, where we used a comprehensive selection of all cases of lung cancer patients without prejudice. Besides, the hospital where we carried out the study is the largest of the 4 hospitals in Marrakech and the only public hospital for the diagnosis and treatment of cancer of all patients from the southern regions.

### 2.2. Material Support

The dependent variable in our study is lung cancer, defined as C34.0-C34.9, which represent malignant neoplasm of bronchus and lung. The subsites of C34.0 through C34.9 are considered part of a single primary site. They represent lesions in the main bronchus excluding Carina (C34.0), in upper, middle, and lower lobes (C34.1, C34.2, and C34.3) and in overlapping lesion (C34.8) of lung, which can also be unspecified (C34.9).

We conducted a survey of patients based on a structured questionnaire and on their recorded medical files which included the history of their disease and the measured diagnosis tests. This questionnaire includes a division of independent variables, which are defined on the basis of ICD-10 codes: sociodemographic data, economic variables, and lifestyle.

#### 2.2.1. Social Criteria

*Educational level* (illiterate, primary, secondary, or higher): this is coded Z55.0, which represents illiteracy and low-level literacy in the section of problems related to education and literacy (Z55); *marital status* (married, divorced, widowed, or single): it is coded Z63.4, which represents disappearance and death of family members, and Z63.5 represents disruption of family by separation and divorce in the section of problems in the relationship with spouse or partner (Z63.0).

#### 2.2.2. Demographic Criteria

*Age and sex*: ages between 28 and 82 years for both women and men; *place of residence*: from five south different regions; *birth rank*: eldest, middle child, or youngest.

#### 2.2.3. Economic Criteria

*Professional status* (active or inactive): it is coded Z56.0, which represents unemployment, unspecified in the section of problems related to employment and unemployment (Z56); *habitat type* (apartment, traditional house, villa, and hut): it is coded Z59.1 and represents inadequate housing in the section of problems related to housing and economic circumstances (Z59); *personal and additional incomes*: it is coded Z59.6, which represents low income in the section of problems related to housing and economic circumstances (Z59).

#### 2.2.4. Lifestyle Criteria

*Body mass index before and after the disease*: obesity, normal, or underweight; *social interaction* (isolated or sociable): it represents living alone and social exclusion and rejection on the basis of illness in the section of problems related to social environment; *sports practice*: it represents limitation of activities due to disability in the section of problems related to life management difficulty; *appetite status*: it represents inappropriate diet and eating habits in the section of problems related to lifestyle; *sleep*: disorder and insomnia; *sedentary behavior*: TV, Internet, and games; b*ehavioral habits* (alcohol consumption, smoking status, and nutrition): it represents tobacco use and alcohol use in the section of problems related to lifestyle and also passive exposure to tobacco smoke presented in the section of problems related to physical environment.

### 2.3. Statistical Analyses

The collected data were coded, captured, and validated. The analysis consisted in a description of the sample according to the sociodemographic characteristics, residence and health conditions of the patients, and their way of life before and after the illness. The description procedure allowed us to describe the distribution of continuous variables and to verify measures of central tendency and dispersion. The correlation test facilitated the determination of the absence or the presence of a significant linear relationship between the continuous variables.

Factors that may influence lung cancer have been the subject of univariate and bivariate analyses to test the possible associations, using the chi-square test to compare qualitative variables with a risk of error granted at 5% and a significance set at *p* < 0.05. The chi-square test was used to test the null hypothesis of the absence of a relationship between two categorical variables and to verify the hypothesis of the independence of these variables. It is based on the comparison of the numbers observed with the theoretical numbers and the calculation of the difference between them. When the difference is very small and does not exceed a theoretical value of chi-square for the corresponding number of degrees of freedom, the two variables are independent of each other. The statistical analyses were performed using the software (IBM SPSS version 20) Statistical Package for the Social Sciences (SPSS Inc., Chicago, Illinois, USA) and expressed as mean ± SD or percentage with significant value.

## 3. Results

### 3.1. Descriptive Characteristics of the Study Population (*N* = 133)

The mean age of patients was 59 ± 9 years.  Table 1 shows that 23% of women and 77% of men in the sample were counted, 35% were professionally active, 83.5% were married, and only 6% had a higher level of education as opposed to 43% who were illiterate. The distribution of the patients according to their place of residence was as follows: Marrakech-Safi (81%), Béni Mellal-Khenifra (7%), Souss-Massa (6%), Drâa-Tafilalet (4%), and Laâyoune-Sakia (2%).

The personal and the additional incomes of the studied population were, respectively, 51% and 64%.  Table 2 shows that 20% of cases had family antecedents of cancer while 4% had personal antecedents of cancer.

A percentage of 32% among patients had cardiovascular diseases, and only 5% of them had rheumatology diseases. Besides, the most remarkable symptoms before the disease diagnosis were coughing, chest pain, dizziness, vomiting, spitting of blood, fever, asthenia, and edema.

### 3.2. Lifestyle: Factors Associated with Lung Cancer

The presence of other diseases and the need for the discovery of the disease were, respectively, strongly related to body mass index (BMI) before the disease with a *p*=0.01/*x*^2^ = 33.15 and a *p*=0.006/*x*^2^ = 32.13.

A number of variables were not associated with the personal history of cancer: social behavior, sedentary behavior, and lifestyle (*p*=0.71/*x*^2^ = 0.13; *p*=0.83/*x*^2^ = 0.04; *p*=0.68*x*^2^ = 0.16, respectively). As we found out in our research, 97% prefer an isolated lifestyle, watching television, and had difficulty in their lifestyle.

The percentage of smokers was particularly high in men (92%) compared with that in women (7%), with *p*=0.00 and *x*^2^ = 89.2. Among daily smokers, 2% of women smoke an average of one pack per day compared to 98% of men who smoke a pack or two a day.

Current smokers (5.3%) plan to quit smoking one day, while nonsmokers (27.1%) express their fear to be subject to lung cancer, most often justifying this feeling by referring to passive smoking; some patients think that breathing the air of cities is as bad for health as smoking cigarettes while others recognize that some people can smoke all their lives without ever getting sick.

We obtained 69% of patients who quit smoking for 10 years before diagnosis, and 28% of them were illiterates compared to 8.8% with a higher level of education. The average age of patients at the initiation of regular smoking is roughly the same for both sexes: 20.3 years for men and 20 years for women. We also found that 45% of patients consume one pack a day and 55% consume it at work.

Habitat type was also related to monthly income with a statistically significant association (*p*=0.01/*x*^2^ = 10.53), and this rate was the lowest (12%) among patients who have no income and who live in huts, while 88% of patients with an income live in a house.

Table 3 shows a strong, statistically significant relationship between the presence of other diseases and the need for the discovery of lung cancer (*p*=0.006/*x*^2^ = 16.15), whereas 20% patients had cough, fever, and digestive pain, 23% had mild tumor and limb pain, and 10% had sputum with blood.

A statistically significant relationship between the type of profession and consumption of canned goods was found among 44% of patients (18.5% farmers, 13.6% workers, and 12.5% craftsmen). Also, we found a very strong statistically significant relationship between the type of profession and consumption of salted and dried meat (quadid). This is found among 44% of patients, including 16.5% farmers, 12.6% workers, and 15.5% craftsmen. In addition, a very strong statistical significance was established between the type of profession and the level of education among 65% of patients: 22.8% farmers, 33.3% unemployed, and 8.9% craftsmen were illiterate.

Among food factors, canned food consumption, oil reuse, and home storage of quadid and khlii are significantly related to lung cancer. While consuming little or no fruit, brine and soft drinks are at greater risk of contracting the disease but not significantly.

In terms of environmental factors, professional toxic exposure increases the risk of lung cancer. Besides, a toxic smoking habit is significantly related to the disease. Also, we found that overweight, sedentary lifestyle, and lack of exercise are independent risk factors, but in the presence of additive effects, they will also be involved in the occurrence of lung cancer.

Finally, there was no statistically significant relationship between the educational level and the consumption of medicinal plants (*p*=0.06/*x*^2^ = 7.48). Thus, there was no statistically significant link between the place of residence and the consumption of brines (*p*=0.06/*x*^2^ = 9.13) in the studied population.

### 3.3. Evolution of Lung Cancer Patients' Living Conditions

**Figure 1:**
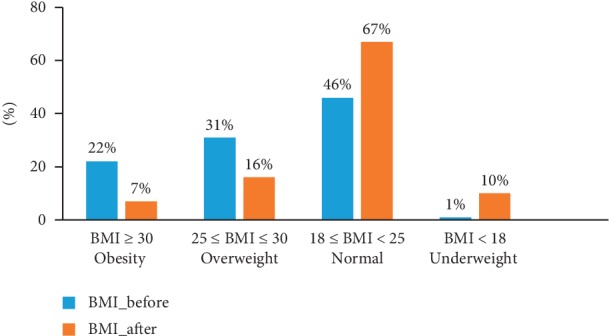
Body mass index (BMI) level before and after lung cancer.

Figure 1 shows that before the disease, 53% of the patients had overweight problems or obesity and 46% had normal weight while only 1% were suffering from underweight. However, after the illness, the frequency of overweight or obesity was only 23%, while 67% of patients were with normal weight and 10% were below normal weight.

Table 4 shows several changes occurring at the level of the patient's living conditions. For example, 98% of them are suffering from insomnia, 93% show a lack of appetite, and 94% are isolated.

## 4. Discussion

We studied the lifestyle of patients who were treated for lung cancer and the factors associated with it. This retrospective study was carried out on a representative sample of patients followed in two departments of the Center of Oncology and Hematology at Mohammed VI University Hospital Center in Marrakech.

As we have worked on a representative sample of 133 patients, we believe that our population can be considered representative of the lung cancer cases that have access to the health system in South Morocco. The absence of cancer records in the southern region during the time of the study did not allow us to know the real status of the different types of cancer.

To our knowledge, this is the first study on the lifestyle of lung cancer patients and the first initiative to assess the level of awareness of certain lung cancer protection parameters in southern Morocco. Lifestyle analysis in lung cancer is affected by some general sources of bias that have become particularly important because of the epidemiological and prognostic specificities of the disease. Indeed, lung cancer is currently classified as small cell lung cancer (SCLC), which is the most dangerous form, accounting for 10–15% of bronchopulmonary cancers, characterized by rapid growth and early metastasis. However, the second type is non-small-cell lung cancer (NSCLC), which includes squamous cell carcinoma and adenocarcinoma and represents nearly 85% of cases. In addition, small-cell bronchial carcinoma most often occurs in central lung localization and primarily affects people with a history of smoking, while the epidemiology of adenocarcinoma is not as conditioned by smoking [14].

The results obtained showed that demographic and socioeconomic characteristics reflected poorly controlled living conditions that contribute to the increase of lung cancer. The key results of this study showed that men seem to be more affected than women with a sex ratio of 3.43. The distribution by age was between 28 and 82 years with an average age of 59 ± 9 years. We found a high frequency for the patients sampled between their forties and their eighties (95%), while the affected population under 40 years was almost inexistent. They also had a low education level, often jobless or working primary and secondary sectors. As far as food factors are concerned, canned food consumption and home storage of quadid and khlii (dried meat cooked with boiling fats) are significantly related to the disease. From another perspective, consuming little or no fruit, brine, and soft drinks may increase the risk of contracting the disease, although it was not significantly proved. The analysis of food consumption showed that there was a significant relationship between the type of profession with the consumption of canned foods and the consumption of salted and dried meat. Regarding the smoking status, we have reached that the factors that led to smoking cessation are education and self-confidence. Besides, we have noticed that the stopping rate seems easier among the most graduates, senior managers, and intermediate professions. On the other hand, we also point out that the low prevalence of smoking among women should not prevent the implementation of prevention actions among women who are considered a potential target of the tobacco industry.

Wintner et al. (2013) found that the average age of patients was 69 years, and 68% of them were men [15]. Contrarily, 59 years was the average age in our study. Also, we found that 97% of men and 100% of women were over the age of 40.

According to Gridelli et al. , lung cancer is frequently diagnosed in elderly patients [16]. The result we reached in our study confirmed that 42% of patients were over the age of 60. After giving up smoking for a period of time ranging from five to nine years, some patients can run just half the risk of lung cancer as compared to those who continue smoking [17]. We obtained 69% of patients who stopped smoking since 10 before diagnosis.

In the majority of Western countries, in particular, the United States and Western Europe, smoking is much more prevalent than in most North African countries such as Morocco, where the tobacco epidemiology has been established more recently [18]. In a multinational study evaluating smoking rates in the Middle East and North Africa, the lowest smoking rate was reported in Morocco (15.3%) [19].

We found that 45% of patients consume one pack per day and 55% consume it at work. And the average age of smoking initiation in the regions of South Morocco was 18 years. A proportion of 50% of smokers had first cigarette consumption before the age of 18. The average age at initiation of smoking in the United States is 15 years, with 87% of smokers having first smoked cigarettes before the age of 18 [20].

A proportion of 91% among 71,000 men were subject to lung cancer death. Also, the death of 77% out of 24,000 women was attributable to smoking [21]. The smoking status of nonsmoking women (21%) indicates that passive inhalation of tobacco smoke causes an increase in the risk of lung cancer in women. There is evidence that about 90% of all lung cancers are related to active inhalation of tobacco smoke [11]. An additional 5% may be associated with exposure to carcinogens at the workplace and radiation (radon) [22]. In the same context, we focused on four major sources leading to cancer covered by Doll and Peto (2015), namely, tobacco (the largest), food and nutrition (the most uncertain), profession (currently, the most controversial), and the most imminent infections [23].

Blot and Tarone [23] found that 20% of lung cancers may be diet-related [21]. While in our study more than 60% of patients were reported to consume food rich in salts (quadid (77%): meat and salty fat; khlii (65%): salty meat; smen (70%): butter salty; preserves (61%)), thereby these results agree with Blot's study.

In Morocco, the awareness of the risks of cancer associated with excessive alcohol consumption is necessary because of the increase in the prevalence of cancer and the consumption of alcohol in recent years. Although alcohol is a risk factor for many types of cancer [24], the frequency of alcohol consumption per month is more than 4 liters for 24% of patients. Beer is the most consumed alcoholic beverage with a percentage of 48.

The personal antecedents of the presence of other diseases most related to lung cancer were cardiovascular, rheumatoid, endocrine, pneumatological, and digestive nature. This result can be explained by the high proportion of these patients whose lifestyle is low.

The subjects with a family antecedent of cancer were less consultant to traditional healers and were most consultant to medical institutions than those who did not have any cancer's family antecedent. This result can be related to the sensitization of patients with a family antecedent of cancer to the severity of the disease and the importance of care and proper medical follow-up.

A study carried out in 2015 found that a number of common clinical problems affect lifestyle and survival in cancer patients, including decreased appetite, frailty, fatigue and decreased activity, weakness, and nausea [25]. This result is consistent with our study, which showed that there is nearly a total absence of sports (98%), lack of appetite (93%), and lack of social interaction after illness (94%). In fact, 98% of the population in our study had insomnia.

We found that 71% weighed less than 70 kg after the illness, which is consistent with the study of Aldige [21] who found that nearly 60% of lung cancer patients lost a lot of weight at the time when they are diagnosed, often due to late diagnosis, and 25% to 50% of these patients are considered malnourished [26].

These important lifestyle factors should be incorporated into the treatment plan at the time of diagnosis and followed throughout the stages of the disease. Other factors such as the stage of cancer or the overall patient health are often used as predictors of survival [21], but in this study, lifestyle at baseline was a better predictor of survival than those two more traditional criteria. About 70% of patients had discovered the disease in less than a year without considering their stage of evolution.

In the study of Najdi et al., the most closely related cancer site to sleep disorders is the pulmonary localization with a rate approaching 85% [27]. This can be explained by the result found in our study; about 98% have been found to suffer from insomnia.

In recent years, based on studies from Norway, South Korea, and different European cities, increased risk of chronic lung diseases was found to be more common in deprived communities and in people with low levels of education and with low socioeconomic status. This can partially be explained by differences in the prevalence of harmful health behaviors, including excessive smoking and poor nutrition [28].

The results of this work encourage us to think about the rapid introduction of measures to promote an active and global follow-up of patients with lung cancer. It should be noted that knowledge of the risk factors for cancer is a determining factor in the process of behavioral change [29].

The strengths of this study lie in its originality being able to highlight the importance of the results of the risk factors and lifestyles of lung cancer patients to improve the management system and care for these patients and develop the most appropriate practices for all health professionals. There are few regional cancer records in the country, and there is no record in the southern regions; hence, the epidemiological study that we conducted lead to prevention actions that are more effective and much less expensive than therapeutic efforts. Moreover, the novelty's pieces of evidence related to this study are manifested in determining the prevalence of lung cancer and its interaction with lifestyle factors for the first time among the Southern region's population that differs from the Northern region's population in their customs, rituals, and behaviors.

Our study, however, has a number of limitations: until 2013, Mohamed VI University Hospital Center represented the only public institution of care for cancer in the region of South Morocco. In addition, the private sector (represented in 2013 by a single private clinic specializing in cancers) is recruiting a very small proportion of cancer cases in South Morocco. Besides, the majority of patients seen at the hospital came from low- and middle-income socioeconomic levels. The smallest proportion of patients from the upper and upper socioeconomic classes seeks care in the few clinics and private hospitals in Marrakech.

Finally, this study shows the need for widespread and systematic public awareness of the lung cancer problem but also the extension of lifestyle surveillance, the expansion of the patient care network, and early detection of lung cancer in the southern region of Morocco.

## 5. Conclusion

The unstable patient living conditions should encourage all health care professionals of the oncology department to work together to take the necessary steps to resolve them.

In response to the purpose of our study, it seems that the use of tobacco and exposure to an environmental factor of risk as tobacco smoke can increase the risk of contracting lung cancer and even be correlated with the disease for people already weakened by food factor. In other words, it seems that food and environmental risk factors would be potentially responsible for the cause of the disease. We also found a regional variability, of exposure to the risk factors of lung cancer, which explains its gradient of incidence in the south of Morocco, in particular, those related to the lifestyle, to the consumption of food and to the professional environment.

The results show that there was a statistically significant difference in the lifestyle, in the physical and nutritional domains. Lifestyle was rated low in emotional status compared to other domains. This could be mainly noticed because of their physical appearance and their psychic suffering, which prevents them from participating in social gatherings and family functions.

The results of this work demonstrate the complexity of the networks of factors that can lead to carcinogenesis and provide also valuable information that could be used to put in place valuable lung cancer prevention strategies in South Morocco. From a global perspective, there is a clear need to closely examine the epidemiology of lifestyle and living conditions before and after lung cancer disease. Knowledge of the risk factors associated with lung cancer and the early prediction of these complications in patients may be useful in clinical practice, particularly, for implementing early preventive measures.

## Figures and Tables

**Table 1:** Distribution of sociodemographic and economic characteristics of patients with lung cancer in the southern regions of Morocco.

Characteristics	%
Age (years)	
< 40	2
Between 40 and 60	55
Between 60 and 80	40
> 80	3
Sex	
Women	23
Men	77
Professional status	
Active	35
Inactive	65
Marital status	
Married or remarried	83
Divorced	7
Widowed	5
Single	5
Place of residence	
Souss-Massa	6
Laâyoune-Sakia	2
Drâa-Tafilalet	4
Marrakech-Safi	81
Béni Mellal-Khenifra	7
Level of study	
Illiterate	43
Primary	25
Secondary	26
Higher	6

**Table 2:** Clinical characteristics and personal and family history of lung cancer disease in the studied population.

Characteristics	%
Personal antecedent of cancer	
Yes	4
No	96
Family antecedent of cancer	
Yes	20
No	80
Presence of other diseases	
Yes	29
No	71
Types of other diseases	
Pneumatological	25
Cardiovascular	32
Digestive	10
Rheumatology	5
Endocrine	28

**Table 3:** The associations between clinical and socioeconomic factors, with the type of profession and with the presence of other diseases in patients with lung cancer.

Variables in association	Controlled variable		
	*χ* ^2^	*p*	% of patients
The type of profession			
Consumption of canned goods	18.79	0.04	44
Consumption of salted and dried meat (Quadid)	21.22	0.02	44
Level of education	94.46	0.00	65
The presence of other diseases			
Fast food and Smen consumption	17.2	0.04	69
Birth rank of the patient	31.8	0.02	66 seniors
Need for the discovery of the disease	16.15	0.006	53

Quadid: dried meat with salt; Smen: butter conserved with salt.

**Table 4:** Changes in behavioral state and lifestyle of patients after lung cancer.

State	% of patients
Insomnia	98
Lack of appetite	93
Social interaction	94 isolated
Absence of sport	98
Sensation of pain	20
Financial issues	21
Weight <70	71

## Data Availability

No data were used to support this study.
